# Triazole-Resistance in Environmental *Aspergillus fumigatus* in Latin American and African Countries

**DOI:** 10.3390/jof7040292

**Published:** 2021-04-12

**Authors:** Agustin Resendiz-Sharpe, Klaas Dewaele, Rita Merckx, Beatriz Bustamante, Maria Celeste Vega-Gomez, Miriam Rolon, Jan Jacobs, Paul E. Verweij, Johan Maertens, Katrien Lagrou

**Affiliations:** 1 Department of Microbiology, Immunology and Transplantation, KU Leuven, 3000 Leuven, Belgium; agustin.resendizsharpe@kuleuven.be (A.R.-S.); rita.merckx@kuleuven.be (R.M.); jjacobs@itg.be (J.J.); johan.maertens@uzleuven.be (J.M.); 2 Excellence Center for Medical Mycology (ECMM), Department of Laboratory Medicine and National Reference Center for Mycosis, University Hospitals Leuven, 3000 Leuven, Belgium; klaas.dewaele@uzleuven.be; 3 Instituto de Medicina Tropical Alexander von Humboldt, Universidad Peruana Cayetano Heredia, Lima 15102, Peru; ana.bustamante@upch.pe; 4 Centro para el Desarrollo de la Investigación Científica, CEDIC, Asunción 1255, Paraguay; mcvegagomez@gmail.com (M.C.V.-G.); rolonmiriam@gmail.com (M.R.); 5 Department of Clinical Sciences, Institute of Tropical Medicine, 2000 Antwerpen, Belgium; 6 Radboud University Medical Center, Department of Medical Microbiology, 6500 HB Nijmegen, The Netherlands; Paul.Verweij@radboudumc.nl; 7 Center of Expertise in Mycology Radboudumc/CWZ, 6500 HB Nijmegen, The Netherlands; 8 Department of Hematology, University Hospitals Leuven, 3000 Leuven, Belgium

**Keywords:** *Aspergillus fumigatus*, antifungal resistance, epidemiology, environment, America, Africa

## Abstract

Triazole-resistance has been reported increasingly in *Aspergillus fumigatus*. An international expert team proposed to avoid triazole monotherapy for the initial treatment of invasive aspergillosis in regions with >10% environmental-resistance, but this prevalence is largely unknown for most American and African countries. Here, we screened 584 environmental samples (soil) from urban and rural locations in Mexico, Paraguay, and Peru in Latin America and Benin and Nigeria in Africa for triazole-resistant *A. fumigatus*. Samples were screened using triazole-containing agars and confirmed as triazole-resistant by the European Committee on Antimicrobial Susceptibility Testing (EUCAST) broth dilution reference method. Isolates were further characterized by *cyp51A* sequencing and short-tandem repeat typing. Fungicide presence in samples was likewise determined. Among *A. fumigatus* positive samples, triazole-resistance was detected in 6.9% (7/102) of samples in Mexico, 8.3% (3/36) in Paraguay, 9.8% (6/61) in Peru, 2.2% (1/46) in Nigeria, and none in Benin. *Cyp51A* gene mutations were present in most of the triazole-resistant isolates (88%; 15/17). The environmentally-associated mutations TR_34_/L98H and TR_46_/Y121F/T289A were prevalent in Mexico and Peru, and isolates harboring these mutations were closely related. For the first time, triazole-resistant *A. fumigatus* was found in environmental samples in Mexico, Paraguay, Peru, and Nigeria with a prevalence of 7–10% in the Latin American countries. Our findings emphasize the need to establish triazole-resistance surveillance programs in these countries.

## 1. Introduction

*Aspergillus fumigatus* is a globally distributed environmental fungus that plays a major role in the decomposition of organic matter. It can adapt to diverse environments and produce vast numbers of spores that assure its survival and dissemination. *A. fumigatus* is usually not a primary human pathogen, but, due to the rise of conditions that require immunosuppressive medications, such as cancer or transplantations, it has become an important agent of opportunistic infections [1]. Recently, *A. fumigatus* was also described as a co-infecting pathogen in patients with severe influenza and COVID-19 [2,3]. Triazole antifungals, such as voriconazole, posaconazole, and isavuconazole, play a crucial role in the prophylaxis and treatment of aspergillus-related diseases [4,5], but increasing reports of triazole-resistance in *A. fumigatus* are a serious concern [6].

Triazole-resistant infections can arise either after prolonged triazole antifungal therapy in patients (patient route of infection) or after exposure to triazole-resistant conidia in the environment (environmental route); the latter has been considered as the main route of infection in patients with invasive aspergillosis [6,7]. It is generally accepted that the development and selection of triazole-resistant *A. fumigatus* in the environment occurs secondary to the exposure of triazole-fungicides (demethylation inhibitors (DMIs)), which are commonly used for crop protection and preservation of materials [8,9]. Environments that contain azole fungicides and allow the fungus to complete all of its growth-cycle stages, such as flower bulbs, green material, and wood chipping waste management sites, are referred to as “hotspots” and have been described to facilitate the emergence, amplification, and spread of mutations that confer triazole-resistance in *A. fumigatus* [9]. The most frequently reported mechanisms conferring triazole-resistance in *A. fumigatus* are mutations in the *cyp51A* gene, a gene involved in the biosynthesis of ergosterol [6,7,10]. Among these, the environmentally associated TR_34_/L98H and TR_46_/Y121F/T289A consisting of a tandem repeat (TR) in the *cyp51A*-promoter region and a single or multiple amino acid substitutions are the most prevalent [6,11]. 

Infections with triazole-resistant *A. fumigatus* have been associated with higher probabilities of therapeutic failure and increased mortality in patients [12,13]. An international expert group has proposed to modify the recommended first-line triazole monotherapy for the treatment of invasive aspergillosis (IA) if environmental triazole-resistance prevalence is >10% to either a combination of a triazole with an echinocandin or liposomal amphotericin B (L-AmB) prior to the availability of susceptibility test results or in the absence of a positive culture [5,14,15].

Although various studies have investigated epidemiological data on triazole-resistance in *A. fumigatus*, the vast majority of countries in the world lack this information as triazole-resistance screening is not routinely performed in these regions [6]. To gain more insight into the current situation of triazole resistance in *A. fumigatus,* we conducted environmental epidemiological studies in countries that lack this information, i.e., Mexico, Peru, and Paraguay in America, and with Benin and Nigeria in Africa.

## 2. Materials and Methods

### 2.1. Environmental Sample Collection

We established collaborations with researchers with a known interest in mycology to collect and screen environmental samples for triazole-resistant *A. fumigatus.* All partners received a detailed protocol for the collection, storage, and shipment of samples in addition to necessary materials. Each collaborator was requested to collect at least 100 environmental samples consisting of 5 g of soil from pre-defined locations in urban and rural areas, such as parks, city and hospital flowerbeds, agricultural fields, greenhouses, gardens, compost, and commercial soil, as stated in our protocol. Environmental samples were collected using a disposable sterile spoon from the surface up to two cm depth where most organic matter is located. Once collected, samples were shipped to our institution for further analysis. Import license for environmental samples was granted by the Federal Agency for Safety of the Food Chain (FASFC) and the Flemish Minister for Environment, Nature and Agriculture, according to the European Union regulations Commission Delegated Regulations 2015/2446, 952/2013 and article 34 of the Royal Decree nr. 7 of 29 December 1992.

### 2.2. Triazole Antifungals Resistance Screening

Two grams from each environmental sample were initially dissolved in 8 mL containing 0.85% Sodium Chloride (NaCl) and 0.1% Tween-20 for analysis. From this, 100 μL was inoculated to each of the following Sabouraud dextrose agar plates containing: no antifungal (control; growth of any fungi), 4 mg/L itraconazole (false positives were previously observed when using 2 mg/L in our pilot study) and 2 mg/L of voriconazole (both from Sigma-Aldrich, St. Louis, MO, USA). As *A. fumigatus* has the ability to grow at high temperatures, agar plates were incubated at 48 °C to restrict the growth of most environmental fungi [16]. Agar plates were monitored for growth for 72 h, which is a short triazole-exposure length with no de novo triazole-resistance induction reports [17]. When growth of *A. fumigatus* complex isolates (based on macro/microscopic characteristics), one isolate from the control and up to 5 from the triazole-containing agar plates were sub-cultured for further investigation. Selected isolates were categorized as *A. fumigatus* sensu-strictu via MALDI-TOF mass spectrometry (Bruker Daltonics, Bremen, Germany) and the Mass Spectrometry Identification platform (MSI) reference spectra database [18]. In case of inconclusive identification, sequencing of the *beta tubulin* gene was performed as described before [10].

### 2.3. Triazole-Resistance Phenotype Determination and Cyp51A Gene Sequencing

Minimal inhibitory concentrations (MIC) of itraconazole, voriconazole, posaconazole, and amphotericin B (all from Sigma-Aldrich, St. Louis, MO, USA) of suspected resistant isolates were determined using the EUCAST (European Committee on Antimicrobial Susceptibility Testing) broth microdilution reference method for filamentous fungi [19]. Triazole-resistant phenotype was confirmed if at least one MIC value was above the established EUCAST resistance clinical breakpoints (voriconazole >1, itraconazole >1, posaconazole >0.25, mg/L) [20]. Only one confirmed triazole-resistant isolate was selected for further analysis from samples harboring multiple resistant isolates with similar phenotypes (± 1 MIC variation) as they were considered the same. MICs from all triazole-resistant and 22 randomly selected susceptible *A. fumigatus* isolates to the azole-fungicide tebuconazole (Sigma-Aldrich, St. Louis, MO, USA) were likewise determined using the EUCAST methodology. Isolates with MIC ≥ 4 mg/L were considered resistant to tebuconazole [21]. Sequencing of the *cyp51A* gene and its promoter region was performed in confirmed triazole-resistant isolates as previously described [22]. 

### 2.4. Genotyping Samples

Genetic relatedness between isolates was determined using two separate multiplex PCRs specific for *A. fumigatus* (M3, M4)*,* each amplifying three short tandem repeats (STRs) of three trinucleotide loci (M3; *STRAf3*-A, B, C primers) and three tetranucleotide loci (M4; *STRAf4*-A, B, C primers) as indicated earlier [23,24]. Briefly, 4 µL of cleaned PCR product was combined with 20 μL mix of HiDi Formamide and GeneScan 500 LIZ size standard (Applied Biosystems, Foster City, CA, USA) for fragment detection on a 96-capillary array ABI3730xl Genetic Analyzer (Applied Biosystems, Foster City, CA, USA). Assignment of STR numbers for each marker was performed using the Peak Scanner Software v1.0 (Applied Biosystems, Foster City, CA, USA). Genetic distance similarity (Euclidean method) and hierarchical relationship (Ward’s minimum variance linkage method; ggplot2 package) based on STRs between our selected isolates and openly available worldwide selected isolates (clinical and environmental) contained within the AfumID-STR profile application were performed using the statistical computing and graphics software R (v4.0.3) [11,25]. Worldwide isolates included at least one susceptible and one triazole-resistant *A. fumigatus* isolate per continent (24 triazole-resistant and 22 wild-types). Based on reported worldwide populations of the AfumID platform, our triazole-resistant isolates were lastly associated with either their described resistant (clade A) or susceptible (clade B) populations (principal component analysis) [11].

### 2.5. Fungicide Concentrations in Environmental Samples

The presence or absence of triazole fungicides in environmental samples (2 g per sample) was determined using multi-residue liquid chromatography-mass spectrometry (Primoris, Zwijnaarde, Belgium). This methodology detects and quantifies >500 pesticides in soil, including triazole fungicides, with a residue limit of detection between 0.01–0.05 mg/kg. All environmental samples from which triazole-*resistant A. fumigatus* was isolated were individually analyzed. Environmental samples where no triazole-resistant *A. fumigatus* was found were combined per country for analysis.

## 3. Results

### 3.1. Triazole-Resistance in A. fumigatus Analysis

We screened 584 environmental samples in total for triazole-resistance in *A. fumigatus*. Overall environmental screening results from all analyzed countries are depicted in Table 1. Detailed analysis per country is as follows:

#### 3.1.1. Mexico

In total, 198 environmental samples (Table 1) were analyzed from 2 regions; 98 samples from Mexico City (urban setting) and 100 samples from Celaya, Guanajuato (urban and rural settings). Growth of any fungi and of *A. fumigatus* complex was observed in 172 samples (87%) and 102 samples (51.5%), respectively. Triazole-resistant *A. fumigatus* sensu-stricto isolates were found in 7 samples, resulting in a resistance prevalence of 6.9% among *A. fumigatus* positive samples. The screened regions in Mexico City and Guanajuato, in addition to the locations of triazole-resistant positive samples are depicted in Figure S1. All resistant isolates were collected in urban sites (7/7), five in Guanajuato (71%) and two in Mexico City (29%). The mycological characteristics of triazole-resistant isolates are depicted in Table 2. Resistance to all tested triazole antifungals was observed in 6 of the 7 isolates (86%), while none were resistant to amphotericin B. *Cyp51A* gene sequencing analysis reported the TR_34_/L98H (5/7, 71.4%) or the TR_46_/Y121F/T289A (1/7, 14.3%) mutations in most isolates, and one isolate (1/7, 14.3%) without mutations. All isolates harboring environmentally associated tandem repeat mutations (6/6) showed cross-resistance to tebuconazole (MIC ≥4 mg/L) with MICs ranging between 8–32 mg/L. No cross-resistance to tebuconazole was observed in the triazole-resistant isolate with wild-type *cyp51A*. Triazole fungicides were not detected in any tested soil samples containing susceptible or resistant isolates.

#### 3.1.2. Paraguay

We screened 85 samples from which growth of any fungi was observed in 62 (73%) and of *A. fumigatus* species complex in 36 samples (42.4%), respectively (Table 1). From this, triazole-resistance was confirmed in three *A. fumigatus* sensu-stricto isolates with a prevalence of 8.3% among *A. fumigatus* complex positive samples. Two isolates harbored amino acid substitutions in the *cyp51A* gene (F46Y, M172V, E427K and F46Y, M172V, N248T, D255E, E427K), and one did not (Table 2). Resistant isolates presented MICs just above the EUCAST resistance clinical breakpoints of >1 mg/L for voriconazole (range of 2–4 mg/L) and were susceptible to posaconazole, itraconazole, and amphotericin B. MICs of tebuconazole ranged between 2–4 mg/L (resistant ≥ 4 mg/L). All samples originated from urban locations. The screened region (Asuncion metropolitan area) and the locations of triazole-resistant positive samples are reported in Figure S2. Triazole fungicides were not detected in any of the analyzed soil samples.

#### 3.1.3. Peru

In Peru, 106 samples were investigated for triazole-resistance (Table 1). Growth of any fungi was detected in 87 samples (82.1%) from which 61 contained *A. fumigatus* species complex isolates (57.5%). We identified six samples with triazole-resistant isolates, resulting in a prevalence among *A. fumigatus* positive samples of 9.8% (6/61). Resistance to all triazole antifungals tested was observed in all isolates (Table 2). Likewise, cross-resistance to the azole-fungicide tebuconazole was detected in all isolates. Resistance to amphotericin B was not found. All six triazole-resistant isolates presented the TR_34_/L98H *cyp51A* gene mutations (100%), with one isolate containing two additional amino acid substitutions (TR_34_/L98H/S297T/F495I). Sampling locations were evenly distributed between rural (3/6, 50%) and urban regions (3/6, 50%) (Figure S3). The presence of two triazole-fungicides, propiconazole and tebuconazole, was confirmed in samples from Peru. Propiconazole was detected in one out of the six triazole-resistant samples (Sample ID 69, 0.012 mg/kg) and tebuconazole in the combined susceptible sample analysis (0.031 mg/kg).

#### 3.1.4. Benin

Samples from 95 locations from Benin were screened for triazole-resistance in *A. fumigatus* (Table 1). In total, 86 samples showed growth of any fungi (90.5%), of which 25 samples contained *A. fumigatus* complex isolates (26.3%). In our analysis, we did not find any triazole-resistant isolates or the presence of triazole fungicides amongst tested samples. Screened regions are reported in Figure S4. 

#### 3.1.5. Nigeria

One hundred samples were screened and analyzed in Nigeria (Table 1). The presence of any fungi and *A. fumigatus* species complex was found in 93 (93.0%) and 46 (46.0%) samples. Only one sample was confirmed to contain a triazole-resistant isolate (urban origin), providing a prevalence of 2.2% (1/46) among culture-positive *A. fumigatus* samples. Mycological characteristics of this isolate are depicted in Table 2. Low levels of resistance were observed in this isolate for all triazole antifungals tested. Similarly, the tebuconazole MIC of 4 mg/L was at the lower limit of the established resistance level (≥4 mg/L). Sequencing of the *cyp51A* gene revealed the amino acid change M172V. The location of the screened regions can be found in Figure S5. No triazole fungicides were present in the resistant or combined susceptible samples. 

### 3.2. Genetic Relatedness Analysis

**Figure 1:**
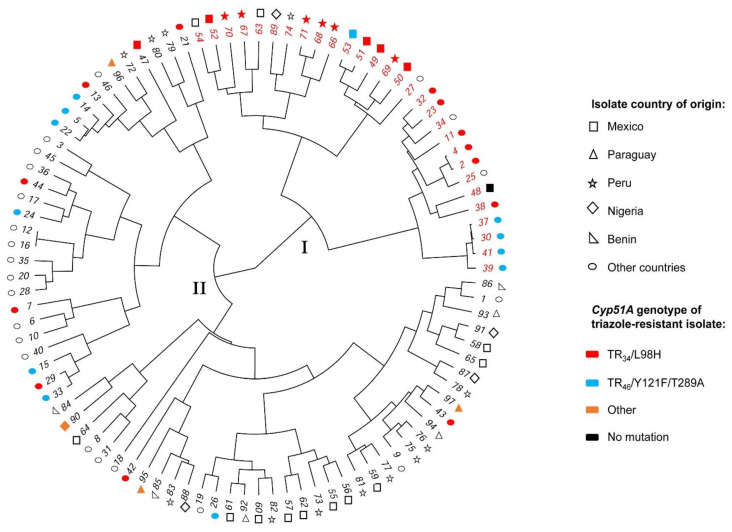
Circular phylogenetic tree depicting the relationship among triazole-resistant and susceptible *Aspergillus fumigatus* isolates. Genetic relationship between triazole-resistant isolates and selected susceptible *Aspergillus fumigatus* isolates from this study and selected publicly available triazole-resistant and susceptible worldwide isolates (short tandem repeat; Euclidian dissimilarity and Ward’s method for row agglomeration). Isolates were clustered into two clades: clade-I (red sample ID number) and clade-II (black sample ID number). Figures represent isolates country of origin. Color inside figures represent the *cyp51A* gene sequencing result (mutations) of triazole-resistant isolates. Abbreviations: ID = identification.

Genetic relationship based on microsatellite profile (STR) between selected *A. fumigatus* isolates from our study (all triazole-resistant) and worldwide isolates from the freely available AfumID-application metadata is depicted in Figure 1. 

Based on our phylogenetic analysis, isolates could be situated into two clades, clade I (red-sample ID number) and clade II (black-sample ID number). Within clade I, we observed a close genetic relationship among most triazole-resistant isolates from Mexico (85%, 6/7) and Peru (100%, 6/6). Triazole-resistant isolates from Nigeria and Paraguay were located in clade II with no strong close genetic relationship between isolates. More triazole-resistant isolates from our study belonged to clade I (12/17, 70%) compared to clade II (5/17, 30%). Based on the interactive web-based tool AfumID metadata, all of our triazole-resistant isolates harboring TR_34_/L98H or TR_46_/Y121F/T289A *cyp51A* gene mutations (12/17) could be associated with their worldwide triazole-resistant population group (Clade A); triazole-resistant isolates harboring other or no *cyp51A* gene mutations (5/17) were associated to clade B were most isolates from the non-resistant population locate (Figure S6). No identical genotype was found between our isolates. Detailed characteristics of genotyped isolates can be found in Figure S7.

## 4. Discussion

Hereby, we report for the first time the recovery of triazole-resistant *A. fumigatus* isolates from environmental samples in three American countries Mexico, Peru and Paraguay, and in the African country of Nigeria with a triazole-resistance prevalence of 6.9% (7/102), 8.3% (3/36), 9.8% (6/61), and 2.2% (1/46), respectively, among *A. fumigatus* culture-positive samples. No triazole-resistant isolates were detected in samples from Benin. 

Epidemiological data on resistance to triazole antifungals in *A. fumigatus* is limited for the American and African continents. In North America, an environmental study from the south-eastern region of the United States (USA) reported the presence of triazole-resistant *A. fumigatus* in 19% (38/200) of analyzed isolates from 8 out of 35 locations (22%) [26]. Two passive surveillance studies performed on clinical isolates reported contrasting triazole-resistant prevalences of 12% (26/220) and 1.4% (20/1356), respectively [27,28]. A lower triazole-resistance prevalence of 0.1% (1/807 patients) was stated in a Canadian surveillance study [29]. In Mexico, the remaining country in the North American region, we found an overall triazole-resistance prevalence of 6.9%. When divided per screened region, we observed in Guanajuato, a region with intense agricultural activities, a higher prevalence of 11.6% (5/43) compared to 3.7% (2/55) in Mexico City. Interestingly, all triazole-resistant samples in both regions originated from urban locations and no fungicide could be detected in our samples. The use of fungicides in Mexico, including azole-fungicides, is high (55% of all used pesticides in Mexico), representing 4% of global consumption with an average-use of 28,601 tons per year (1.45 kg/used-hectare) [30,31]. Amongst authorized agricultural fungicides, four groups that compromise the production of ergosterol by inhibition of the cyp51A enzyme (sterol biosynthesis inhibitors (SBI)) have been described: demethylation inhibitors (DMIs; SBI class I), amines (SBI class II), keto-reductase inhibitors (SBI class III) and squalene monooxygenases (SBI class IV) [32]. Based on a homology cyp51A protein model of *A. fumigatus*, it was reported that most compounds from the DMIs group adopt similar positions upon docking in the protein’s active site as those observed for medical triazoles [33]. The core structure of the DMIs propiconazole and bromuconazole (long-tailed fungicides) were most similar to itraconazole and posaconazole (long-tailed antifungals), while tebuconazole and epoxiconazole (short-tailed fungicides) relate more to voriconazole (short-tailed antifungal) [33,34]. The structure of the DMIs difenoconazole was different as it docked into the access channel of medical azoles, a region associated with conferring triazole-resistance. These five fungicides showed complete loss of in vitro activity against resistant isolates and their use might play an important role in environmental cross-resistance selection to medical triazoles.

In Mexico, tebuconazole is the most sold fungicide, followed by difenoconazole, propiconazole and epoxiconazole, with an average-use between 0.1–0.3 kg/used-hectare [35,36]. However, we could not detect the presence of any DMIs in any of our samples from Mexico. Nonetheless, the majority of triazole-resistant isolates (6/7) showed cross-resistance to tebuconazole. These isolates were most likely selected in the environment in locations where DMIs are present, which may be more prevalent in Guanajuato and spread through these regions. Our reported overall triazole-resistance prevalence of 6.9% is similar to the 8.3% described in a tertiary care facility located in Mexico City (2/24) [37], but higher than the one observed in our screened regions in Mexico City (3.7%), which included the location of this facility. However, tertiary care facilities in Mexico generally treat patients from different regions. However, as the number of samples from their study was small, we cannot infer any conclusions.

In the South American region, our reported triazole-resistance prevalence in Paraguay (8.3%) and in Peru (9.8%) is higher to one reported from Brazil (4.5%, 2/44 cases) but much lower than the ones reported from two environmental studies from Colombia of 47.1% (8/17) and 44.1% (15/34), respectively [38,39,40]. However, due to the low sample numbers in these studies, it is unclear how representative these prevalence data are. The triazole-resistant isolates from Colombia were mainly found in flower fields and vegetable crop zones; therefore, their reported prevalence might be more representative of hotspots with an increased selection of resistant isolates and not for the global situation in this country. According to the latest reports of the Food and Agriculture Organization of the United Nations (FAO) [31], Colombia has the highest fungicides-use per country in that region with an average-use of 7214 tons (13.5 kg/used-hectares) compared to the used in Paraguay (4556 tons; 3.82 kg/used-hectare) and Peru (1189 tons; 1.0 kg/used-hectare). In Peru, currently only tebuconazole and propiconazole have national use registration [41,42]. From these, tebuconazole is the most used with and average-use of 0.10–0.16 kg/used-hectare [35,41]. In Paraguay, several DMIs are authorized for agricultural use [43], yet tebuconazole and propiconazole are the most frequent with a 0.10 and 0.12 kg/used-hectare average-use, respectively [35,44]. The presence of higher concentrations (0.15–0.69 mg/kg) and/or combination of detected azole-fungicides (four) in the Colombian samples compared to our detected concentrations in Peru (propiconazole 0.012, tebuconazole 0.031 mg/kg) and none in Paraguay might play a role in the selection and the observed differences in prevalence. A recent prospective study from Peru, reported a triazole-resistance prevalence of 2.1% among clinical isolates (3/143), which is lower than our reported prevalence of 9.8% [45]. Half of the isolates included in their study were cultured from patients with a history of pulmonary tuberculosis with suspected chronic pulmonary aspergillosis. Colony testing of up to five colonies is recommended to increase sensitivity for triazole-resistance detection [5]. Within this study, only one *A. fumigatus* colony was analyzed from each sample, increasing the risk of selecting a susceptible isolate in patients co-infected with triazole-resistant isolates [46], which could contribute to the observed differences in prevalence. 

Among our two screened countries in Western Africa, we found only one triazole-resistant isolate in Nigeria, resulting in a low prevalence of 2.2% (1/46) and none in Benin (0/25) amongst *A fumigatus* positive samples. Triazole-resistance in *A. fumigatus* in Africa has been reported in Burkina Faso in Western Africa and in Tanzania and Kenya in Eastern Africa, countries characterized by extensive farming, greenhouses, and/or horticultural practices. As reported in Nigeria, the study from Burkina Faso likewise described a low environmental triazole-resistance prevalence of 2.0% (1/51) amongst *A. fumigatus* positive samples [47]. In contrast, an environmental study performed in urban and rural locations in the Eastern African country of Tanzania reported triazole-resistant *A. fumigatus* in 37.0% (10/27) of their *A. fumigatus* positive samples [48]. In Kenya, a study conducted in soil samples from agricultural sites reported 27.1% (13/48) to harbor resistant *A. fumigatus* isolates to at least one triazole-antifungal [49]. 

Within our analyzed samples, we did not detect the presence of any fungicides. DMIs fungicides are authorized in all the stated African countries. However, information concerning the average-use of triazole-fungicides in these countries could not be found, yet the use of pesticides, including fungicides, have been reported in Kenya, Tanzania, and Nigeria, with a use per area of cropland of 0.43, 0.1, and 0.1 kg/used-hectare; in Benin and Burkina Faso, studies refer to the use of pesticides, but no data concerning used amounts per area could be found [31,50,51,52]. The use of pesticides seems comparable between these countries. However, the known extensive use of fungicides in the flower and horticultural industry in Kenya and in the farming regions of Tanzania likely established environments (hot spots) that enabled the selection and dissemination of triazole-resistant isolates in the immediacies of the screened locations in these countries that could explain the increased prevalences observed [49,53]. Whether our observed low prevalence of triazole-resistant *A. fumigatus* isolates of 2.2% in Nigeria or its absence in Benin represents current situations or is secondary to sampling locations will require further investigation.

With regard to *cyp51A* gene mutations, the most commonly reported environmentally associated TR_34_/L98H mutations were the most prevalent resistance mechanism in our triazole-resistant isolates from Peru (100%, 6/6) and Mexico (71%, 5/7), with two isolates harboring two additional substitutions (S297T, F495I). Similarly, this is the only mutation reported in clinical isolates from Peru (one isolate) and Mexico (two isolates), supporting the link between environment-patient transmission in these countries. Isolates harboring this mutation have also been reported in the USA, Brazil, and Colombia [26,27,28,38,39,40]. Interestingly, three samples harboring TR_34_/L98H triazole-resistant isolates from Mexico (two samples) and Peru (one sample) were retrieved from locally produced commercially available compost. The prevalence of triazole-resistant *A. fumigatus* in commercial compost was the highest amongst all screened locations in Mexico (15%; 2/13 compost-samples) but not in Peru (5%, 1/19 compost-samples), where agricultural locations were (14.3%, 3/21 agricultural-samples). We could not detect the presence of fungicides in any of these three samples that could explain our finding, yet this niche may pose a potential risk for triazole-resistance dissemination and requires further investigation. We found one isolate harboring the TR_46_/Y121F/T289A mutation in Mexico, the second most commonly reported environmentally-associated mutation. This mutation has also been reported in Argentina, Colombia, and the USA [38,39,40,54]. Most of our isolates harboring the TR_34_/L98H and TR_46_/Y121F/T289A mutations showed a close genetic relationship (clade I; 92%,11/12) and could be likewise associated to the triazole-resistant population clade (A-Blue) within the AfumID-application metadata (worldwide isolates), suggestive of a probable shared common ancestor among these isolates and a global distribution [11].

The remaining *cyp51A* mutations were the M172V in one isolate from Nigeria and a combination of the F46Y, M172V, N248T, D255E, and E427K in two isolates from Paraguay. In the two isolates from Paraguay, only the MIC value for voriconazole was elevated (2 mg/L) and fell into the area of technical uncertainty (ATU). These amino acid substitutions are located in the periphery of the protein and not in regions conferring azole-resistance and have been reported, as well in susceptible isolates, inferring these variances as *cyp51A* gene polymorphisms [7,10,24,55,56,57,58]. Regardless, our genetic relatedness analysis located isolates harboring these mutations in clade II where broad genetic diversities and most susceptible isolates were found (87.5%, 49/56), implying that these isolates were most likely selected independently in the environment and did not originate from a common ancestor. 

The finding of triazole-resistant *A. fumigatus* spores in the environments of Mexico, Nigeria, Paraguay and Peru represents a public health concern in these countries. It is recommended that in regions with known triazole-resistance, all clinically relevant isolates should be subjected to susceptibility testing, but this is not the case in most centers in these countries [5]. Agar-based screening methods for triazole-resistance can be helpful to select colonies for susceptibility testing [59], reducing cost and hands-on time compared to carrying out susceptibility testing to all isolates. 

Our findings highlight that triazole resistance in environmental *A. fumigatus* is a concern in several American countries supporting the need to establish local and national continuous surveillance programs in clinical and environmental settings of triazole-resistance in *A. fumigatus* in addition to rapid diagnostics capabilities and awareness amongst professionals. It is essential to closely monitor the current and evolving triazole-resistance situation to establish local recommendations for the initial treatment of invasive aspergillosis. This study provides further evidence that triazole resistance in *A. fumigatus* is a global concern that requires epidemiological surveillance studies in countries still lacking this information.

## Figures and Tables

**Table 1:** Environmental screening of triazole resistant *Aspergillus fumigatus (A. fumigatus)* per country.

Country	Samples (No.)	% of Total Samples	% of *A. fumigatus* Positive Culture Samples
**Mexico**			
Total samples	198		
Growth any fungi	172	87.0	
*A. fumigatus species*	102	51.5	
Triazole-resistant *A. fumigatus*	7	3.5	6.9
*Cyp51A* gene mutation	6	3.0	5.9
No mutation *cyp51A* gene mutation	1	0.5	1.0
**Paraguay**			
Total samples	85		
Growth any fungi	62	73	
*A. fumigatus species*	36	42.4	
Triazole-resistant *A. fumigatus*	3	3.5	8.3
*Cyp51A* gene mutation	2	2.4	5.6
No mutation *cyp51A* gene mutation	1	1.2	2.8
**Peru**			
Total samples	106		
Growth any fungi	87	82.1	
*A. fumigatus species*	61	57.5	
Triazole-resistant *A. fumigatus*	6	5.7	9.8
*Cyp51A* gene mutation	6	5.7	9.8
No mutation *cyp51A* gene mutation	0	0.0	0.0
**Benin**			
Total samples	95		
Growth any fungi	86	90.5	
*A. fumigatus species*	25	26.3	
Triazole-resistant *A. fumigatus*	0	0.0	0.0
*Cyp51A* gene mutation	0	0.0	0.0
No mutation *cyp51A* gene mutation	0	0.0	0.0
**Nigeria**			
Total samples	100		
Growth any fungi	93	93	
*A. fumigatus species*	46	46	
Triazole-resistant *A. fumigatus*	1	1.0	2.2
*Cyp51A* gene mutation	1	1.0	2.2
No mutation *cyp51A* gene mutation	0	0.0	0.0

**Table 2:** Mycological characteristics of triazole-resistant *Aspergillus fumigatus* environmental isolates.

Country—Regions:	Sample ID Number	EUCAST MIC (mg/L) ^1^					*cyp51A* Gene Mutation	Location ^3^
		AMB	VCZ	POS	ITC	TEB ^2^		
**Mexico**								
Guanajuato	47	1	4	0.5	>8	8	TR_34_/L98H	Flower bed (U)
Guanajuato	48	1	4	0.5	16	0.5	No mutation	Park (U)
Guanajuato	49	0.5	2	1	>16	8	TR_34_/L98H	Commercial compost (U)
Guanajuato	50	1	4	0.5	>16	8	TR_34_/L98H/S297T/F495I	Commercial compost (U)
Guanajuato	51	1	16	2	>16	32	TR_34_/L98H	Flower bed (U)
Mexico City	52	0.5	4	0.5	>16	16	TR_34_/L98H	Greenhouse (U)
Mexico City	53	0.5	>16	0.5	1	16	TR_46_/Y121F/T289A	Flower bed (U)
**Paraguay**								
Asunción	95	0.5	2	0.25	1	4	F46Y, M172V, E427K	Flower bed (U)
Asunción	96	1	4	0.25	1	2	No mutation	Flower bed (U)
Asunción	97	1	2	0.25	1	4	F46Y, M172V, N248T, D255E, E427K	Flower bed (U)
**Peru**								
Lima City	66	1	2	1	>16	8	TR_34_/L98H/S297T/F495I	Agricultural field (R)
Lima City	67	1	8	1	>16	16	TR_34_/L98H	Agricultural field (R)
Lima City	68	0.25	8	1	>16	16	TR_34_/L98H	Park (U)
Lima City	69	0.5	8	1	>16	16	TR_34_/L98H	Flower bed (U)
Lima City	70	0.5	4	0.5	>16	32	TR_34_/L98H	Agricultural field (R)
Lima City	71	0.5	8	1	>16	64	TR_34_/L98H	Commercial compost (U)
**Nigeria**								
Lagos Ibadan	90	1	2	0.25	4	4	M172V	Flower bed (U)

^1^ Minimal inhibitory concentration (MIC) determined by the European Committee on Antimicrobial Susceptibility Testing (EUCAST) broth microdilution reference method for filamentous fungi. Triazole-resistant phenotype was confirmed if at least one MIC value was above the EUCAST resistance clinical breakpoint (voriconazole > 1, itraconazole >1, posaconazole >0.25, mg/L). ^2^ Triazole fungicide tebuconazole; Resistance ≥4 mg/L. ^3^ Location: Urban area (U), rural area (R). Abbreviations: ID = identification, AMB = amphotericin B, VCZ = voriconazole, POS = posaconazole, ITC = itraconazole, TEB = tebuconazole.

## Data Availability

Study data is contained within the article or Supplementary Material.

## Supplementary Materials

The following are available online at https://www.mdpi.com/article/10.3390/jof7040292/s1, Figure S1: Regions of Mexico screened for triazole-resistant *Aspergillus fumigatus*, Figure S2: Paraguay environmental screening regions, Figure S3: Peru’s screened region for triazole-resistant *Aspergillus fumigatus*, Figure S4: Screened regions of Benin for triazole-resistant *Aspergillus fumigatus*, Figure S5: Environmental screening regions for triazole-resistant *Aspergillus fumigatus* in Nigeria, Figure S6: Principal component analysis of triazole-resistant *Aspergillus fumigatus* isolates, Figure S7: Genetic relationship and characteristics of selected *Aspergillus fumigatus* isolates according to short tandem repeats typing.
