# Supplementary material for: Triazole-Resistance in Environmental Aspergillus fumigatus in Latin American and African Countries
Source: J Fungi (Basel). 2021 Apr 12;7(4):292. doi: 10.3390/jof7040292 (PMC8070258; doi:10.3390/jof7040292)
Supplement: Supplementary file 1 [file jof-07-00292-s001.pdf]

Supplementary Material

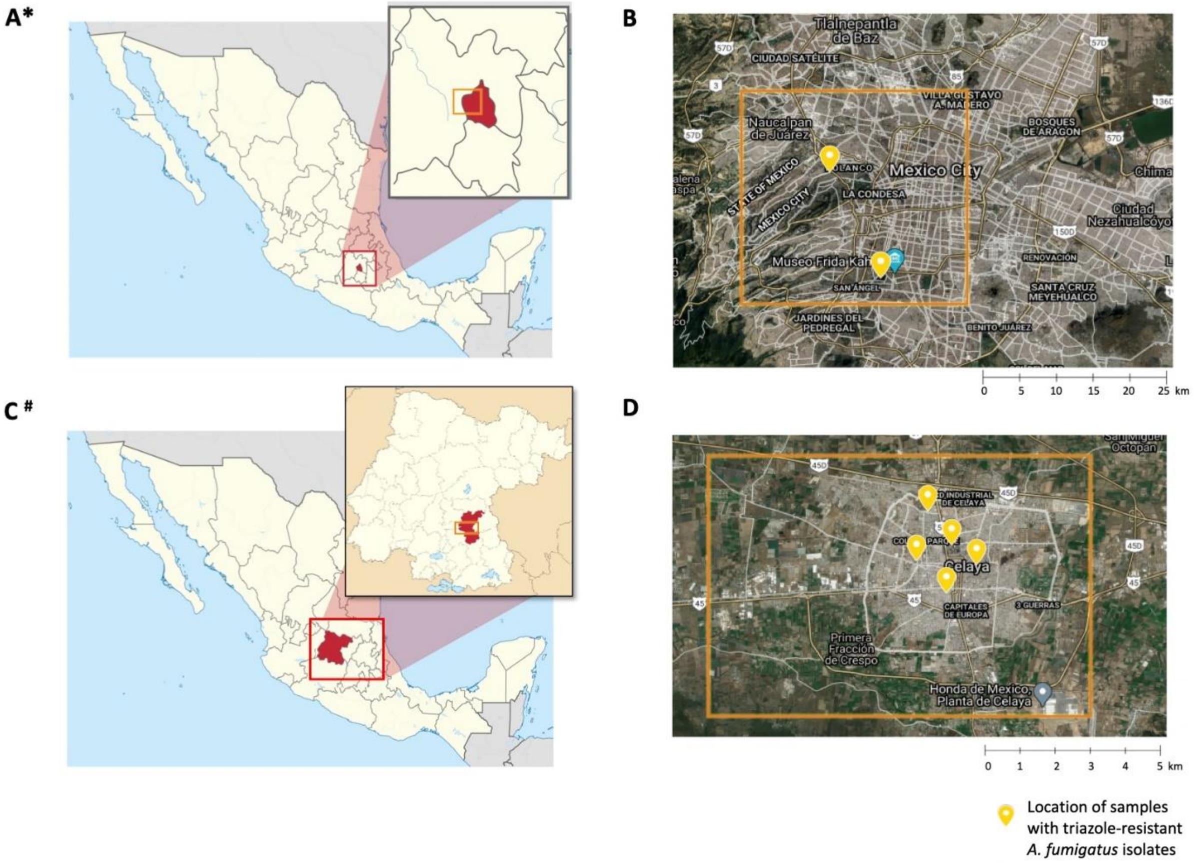

Figure S1.- Regions of Mexico screened for triazole-resistant *Aspergillus fumigatus*. Representative images portraying Mexico's geographical distribution by states accentuating in red the analyzed region of **A)** Mexico City and **C)** the state of Guanajuato; and screened locations situated within the yellowed-squared area. The location of samples with triazole-resistant *A. fumigatus* isolates in the metropolitan area of **B)** Mexico City and **D)** Guanajuato in the screened regions are marked in yellow.

* This vector image includes elements that have been taken or adapted from the publicly available file: Mexico (city) in Mexico (zoom).svg (by TUBS), August 2011, retrieved 18:17, January 1, 2021, from https://commons.wikimedia.org/wiki/File:Mexico_(city)_in_Mexico_(zoom).svg#globalusage.

# This vector image includes elements that have been taken or adapted from the publicly available file: Guanajuato in Mexico (location map scheme).svg (by TUBS), August 2011, retrieved 18:17, January 1, 2021, from https://commons.wikimedia.org/wiki/File: Mexico_(city)_in_Mexico_(zoom).svg#globalusage.

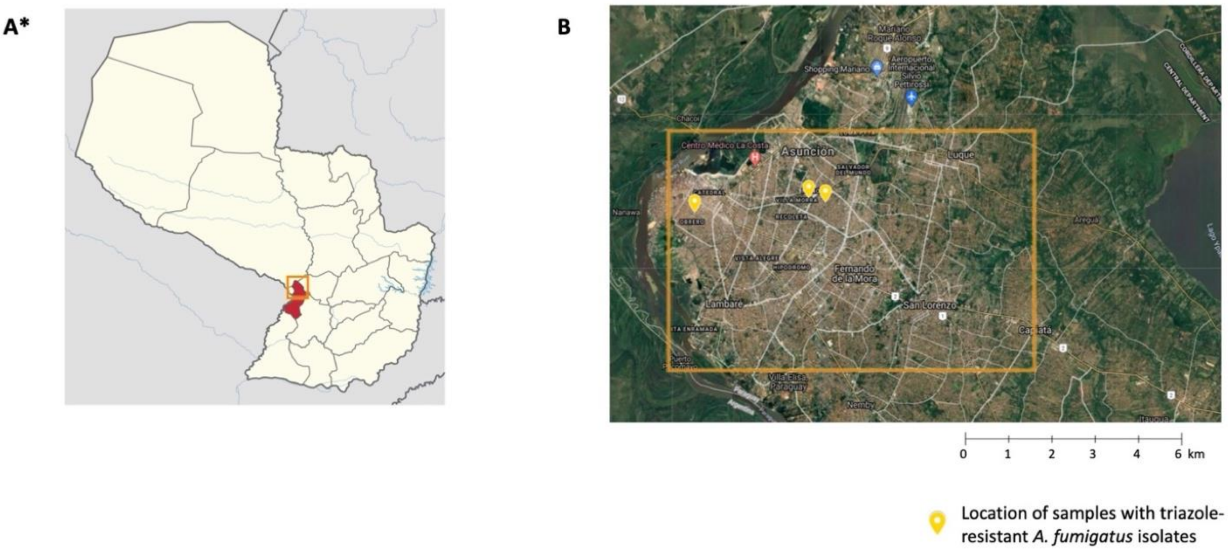

Figure S2.- Paraguay environmental screening regions. **A)** Paraguay's administrative regions accentuating in red the capital district in addition to the screened region (yellow-squared area). **B)** The location of samples with triazole-resistant *A. fumigatus* isolates within the screened regions are marked in yellow.

* This vector image includes elements that have been taken or adapted from the publicly available file: Central in Paraguay.svg (by TUBS), November 16, 2011. retrieved 14:38, January 12, 2021, from https://commons.wikimedia.org/wiki/File:Central_in_Paraguay.svg.

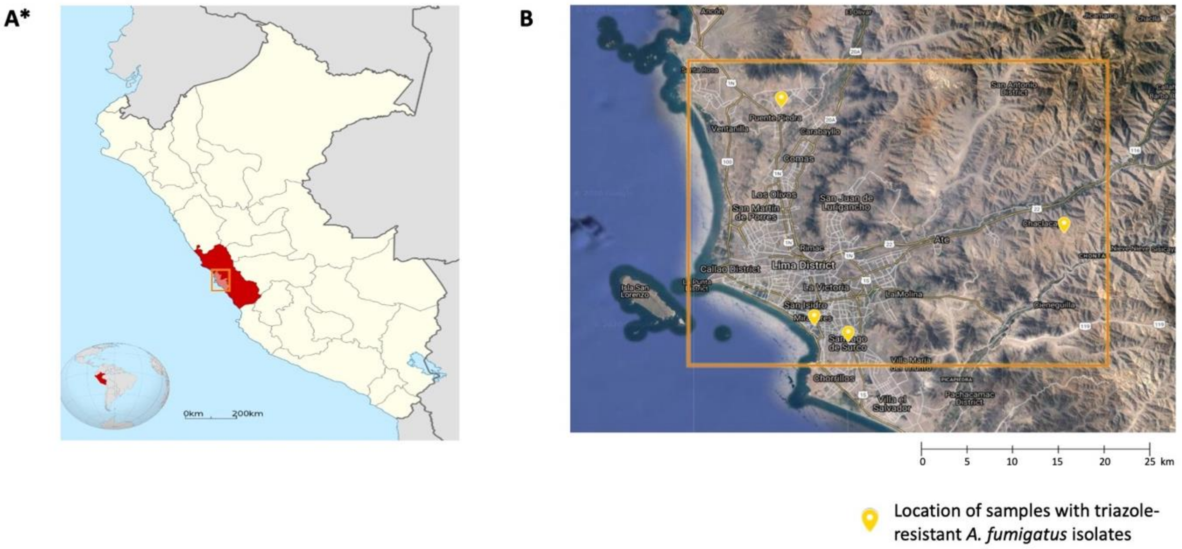

Figure S3.- Peru's screened region for triazole-resistant *Aspergillus fumigatus*. **A)** Representative image portraying the departmental regions of Peru. Depicted in red are the department of Lima (dark-red) and the metropolitan area of the city of Lima (light-red). The screened region is contained within the squared region (yellow-squared area). **B)** Location of samples with triazole-resistant *A. fumigatus* isolates (yellow-marker) within the screened region (square).

* This vector image includes elements that have been taken or adapted from the publicly available file: Peru_-_(Template).svg

(by Huhsuqu)., July 2009, retrieved 18:17, January 10, 2021 from https://commons.wikimedia.org/wiki/File:Peru_-_Lima_Department_(locator_map).svg?uselang=fr.

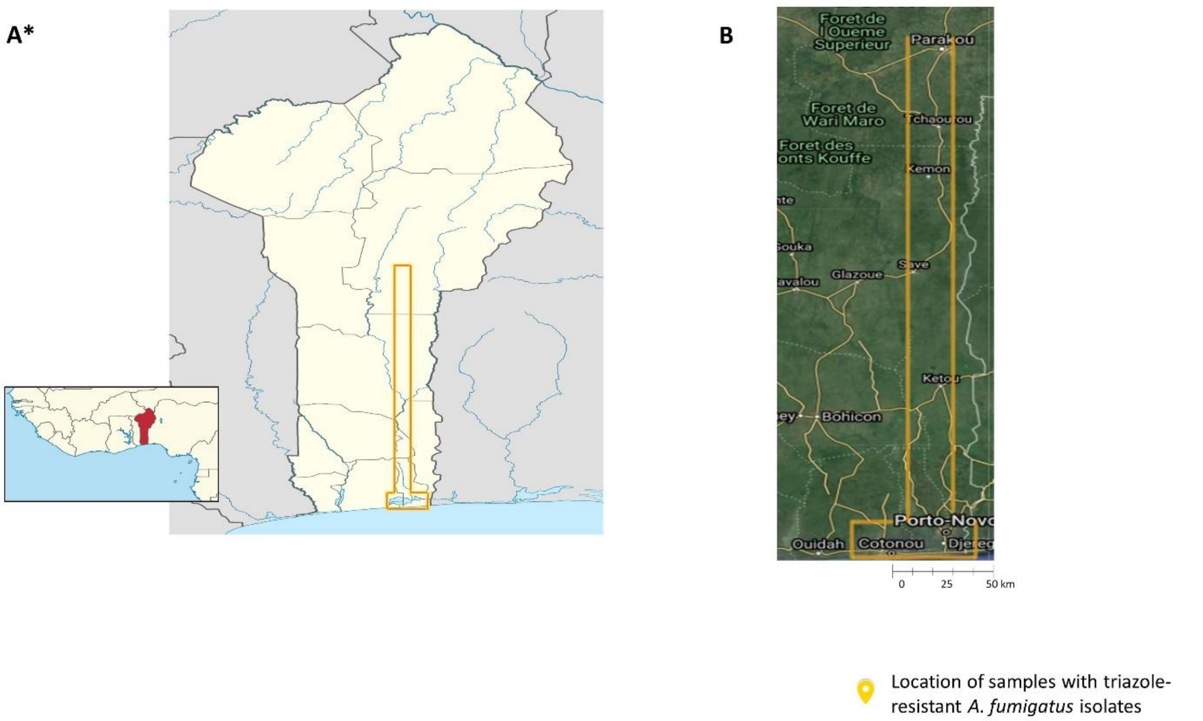

Figure S4.- Screened regions of Benin for triazole-resistant *Aspergillus fumigatus*. A) Depictive image of Benin's departments showing the screened region located within the yellow-lined area with **B)** an enlarged view of sampling location of screened regions.

* This vector image includes elements that have been taken or adapted from the publicly available file: Benin in its region.svg (by TUBS), May 2011, from https://commons.wikimedia.org/wiki/File:Benin_in_its_region.svg.; and the file: Benin location map.svg (by NordNordWest), January 02, 2009., from https://es.m.wikipedia.org/wiki/Archivo:Benin_location_map.svg., retrieved at 19:27, January 11, 2021.

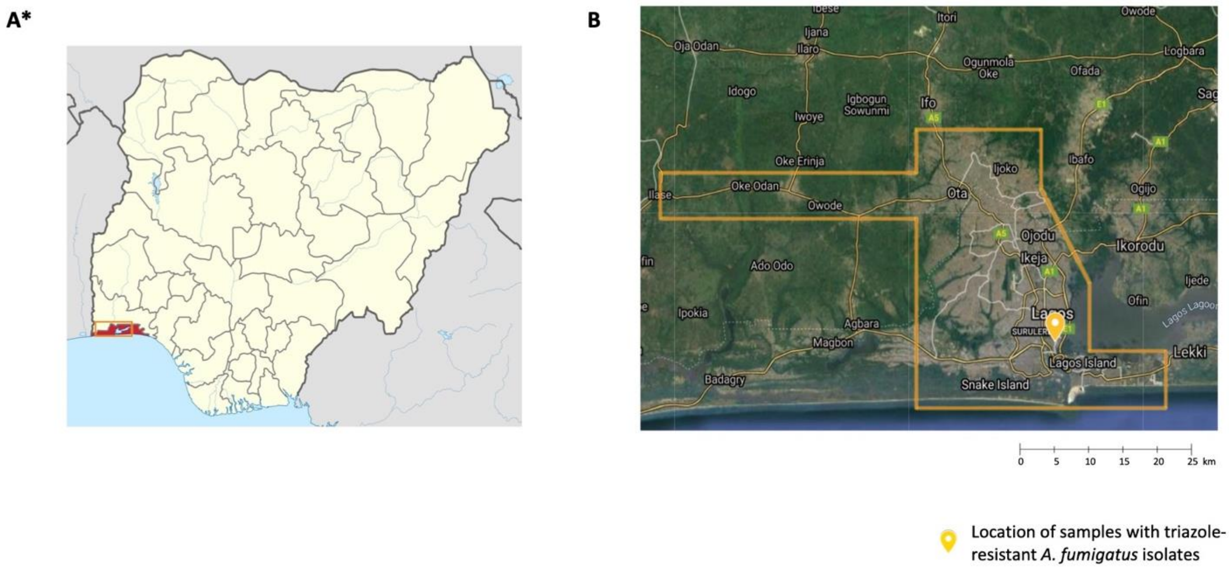

Figure S5.- Environmental screening regions for triazole-resistant *Aspergillus fumigatus* in Nigeria. **A)** Representative image portraying Nigeria's states depicting the state of Lagos in red and the screened region within the lined area (yellow line). **B)** Location of samples with triazole-resistant *A. fumigatus* isolates (yellow-marker) within the screened region (yellow-lined area).

* This vector image includes elements that have been taken or adapted from this file: Nigeria location map.svg (by Uwe Dedering)., CC BY-SA 3.0, https://commons.wikimedia.org/w/index.php?curid=17436041

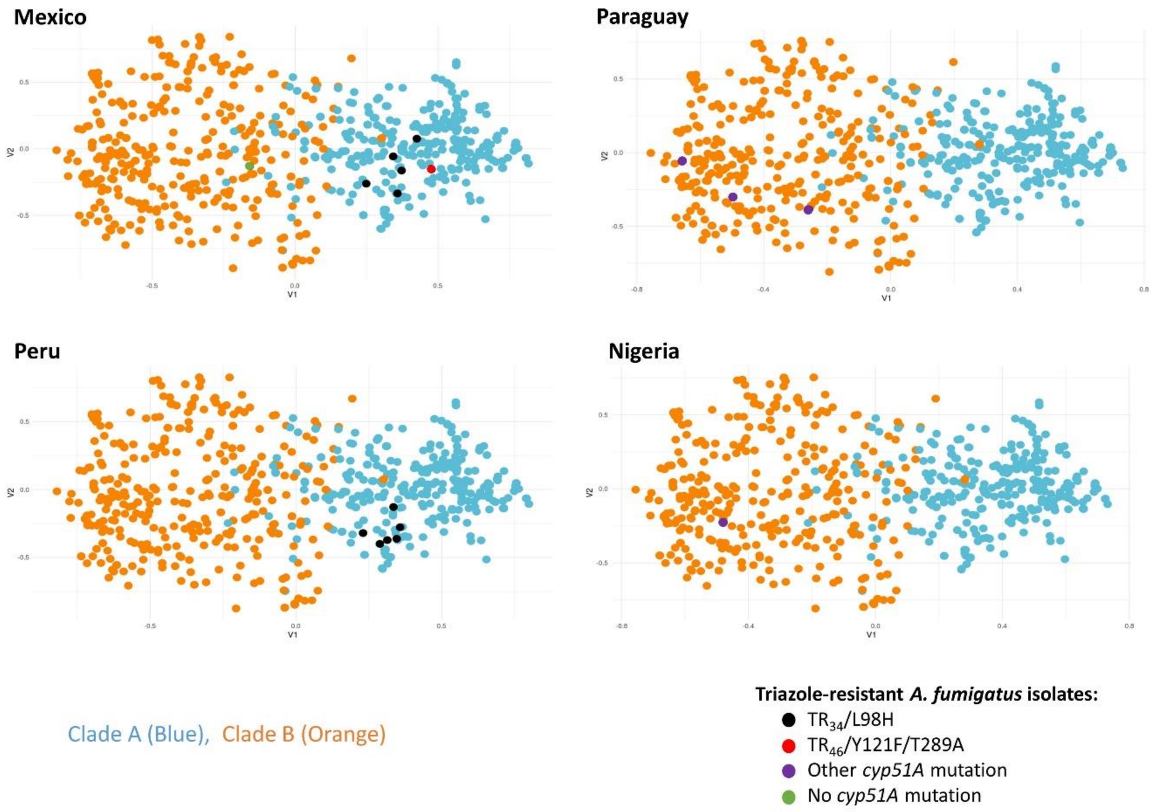

Figure S6.- Principal component analysis of triazole-resistant *A. fumigatus* isolates. Genetic relatedness analysis of triazole-resistant *A. fumigatus* isolates from Mexico, Paraguay, Peru, and Nigeria based on short tandem repeat profile according to the interactive web-based tool AfumID metadata application (principal component analysis). Clade A (blue) represents the population where the majority of analyzed worldwide triazole-resistant isolates and of the cyp51A alleles TR$_{34}$/L98H and TR$_{46}$/Y121F/T289A locate. Clade B (orange) portrays the population where most susceptible (wild-type) isolates are distributed.

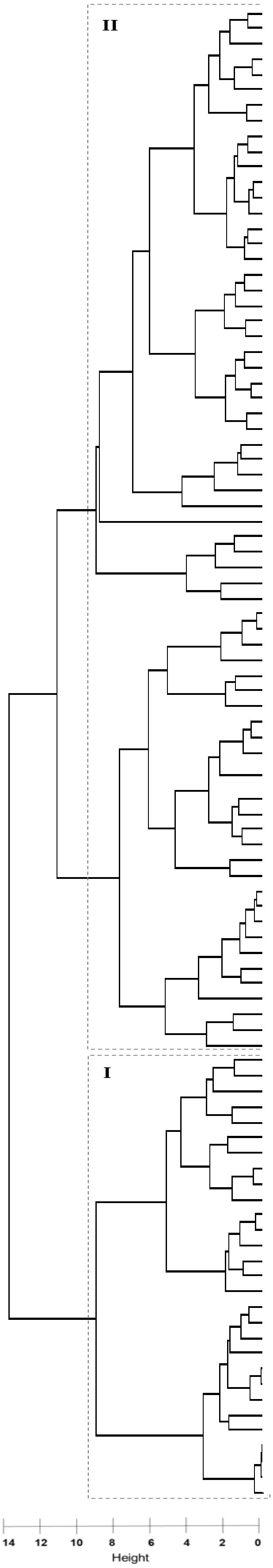

Sample ID number	Short Tandem repeat						Origin	Country	Triazole phenotype	*Cyp51A* sequencing result
	3A	3B	3C	4A	4B	4C				
86	32	21	38	8	9	6	Environmental	Benin	Susceptible	WT
1	26	23	34	9	9	5	Environmental	Australia	Susceptible	WT
93	35	21	42	9	6	10	Environmental	Paraguay	Susceptible	WT
91	27	16	34	6	8	8	Environmental	Nigeria	Susceptible	WT
58	26	16	30	7	7	9	Environmental	Mexico	Susceptible	WT
65	25	14	39	9	5	8	Environmental	Mexico	Susceptible	WT
87	28	16	36	13	6	8	Environmental	Nigeria	Susceptible	WT
78	34	16	37	14	7	5	Environmental	Peru	Susceptible	WT
97	25	18	39	11	9	6	Environmental	Paraguay	Resistant	F46Y, M172V, N248T, D255E, E427K
43	29	14	40	10	9	5	Environmental	India	Resistant	TR$_{34}$/L98H
94	28	17	42	9	12	6	Environmental	Paraguay	Susceptible	WT
76	28	12	36	10	10	7	Environmental	Peru	Susceptible	WT
75	26	14	36	9	10	8	Environmental	Peru	Susceptible	WT
9	25	11	32	8	10	7	Clinical	Japan	Susceptible	WT
77	20	16	39	13	10	7	Environmental	Peru	Susceptible	WT
59	18	14	40	11	9	9	Environmental	Mexico	Susceptible	WT
81	20	16	38	10	11	10	Environmental	Peru	Susceptible	WT
56	34	7	32	7	7	8	Environmental	Mexico	Susceptible	WT
55	38	5	40	6	7	5	Environmental	Mexico	Susceptible	WT
73	28	7	37	4	9	7	Environmental	Peru	Susceptible	WT
62	21	8	33	9	6	5	Environmental	Mexico	Susceptible	WT
57	23	7	32	6	7	5	Environmental	Mexico	Susceptible	WT
82	40	8	35	10	7	10	Environmental	Peru	Susceptible	WT
60	35	10	36	11	9	9	Environmental	Mexico	Susceptible	WT
92	42	10	34	8	9	8	Environmental	Paraguay	Susceptible	WT
61	40	7	32	7	9	8	Environmental	Mexico	Susceptible	WT
26	39	8	49	8	10	9	Environmental	Colombia	Resistant	TR$_{46}$/Y121F/T289A
19	41	11	48	10	9	8	Clinical	The Netherlands	Susceptible	WT
88	47	17	34	10	10	5	Environmental	Nigeria	Susceptible	WT
83	44	13	37	10	12	8	Environmental	Peru	Susceptible	WT
85	45	18	36	9	10	12	Environmental	Benin	Susceptible	WT
95	55	15	37	16	12	6	Environmental	Paraguay	Resistant	F46Y, M172V, E427K
42	68	9	8	8	9	11	Clinical	Germany	Resistant	TR$_{34}$/L98H
18	39	11	48	10	26	8	Clinical	The Netherlands	Susceptible	WT
31	31	29	25	14	9	8	-	Denmark	Susceptible	WT
8	35	33	23	10	11	8	Clinical	Brasil	Susceptible	WT
64	36	40	31	7	9	9	Environmental	Mexico	Susceptible	WT
90	16	24	34	4	11	8	Environmental	Nigeria	Resistant	M172V
84	22	29	40	6	6	5	Environmental	Benin	Susceptible	WT
33	32	9	10	8	14	10	-	Denmark	Resistant	TR$_{46}$/Y121F/T289A
29	32	11	11	8	14	10	Environmental	Colombia	Resistant	TR$_{34}$/L98H
15	33	11	22	8	14	10	Environmental	Tanzania	Resistant	TR$_{46}$/Y121F/T289A
40	26	19	17	10	16	8	Clinical	Germany	Susceptible	WT
10	8	15	7	10	11	10	Clinical	kuwait	Susceptible	WT
6	9	8	10	8	9	10	Clinical	Ireland	Susceptible	WT
7	10	10	10	8	10	20	Environmental	Ireland	Resistant	TR$_{34}$/L98H
28	29	12	26	10	10	5	Environmental	Colombia	Susceptible	WT
20	27	9	28	10	9	5	Environmental	The Netherlands	Susceptible	WT
35	25	11	22	8	8	5	-	France	Susceptible	WT
16	23	9	8	9	9	5	Environmental	Tanzania	Susceptible	WT
12	23	9	8	9	9	5	-	Romania	Susceptible	WT
24	43	9	10	12	9	9	Environmental	The Netherlands	Resistant	TR$_{46}$/Y121F/T289A
17	38	9	10	8	10	8	Clinical	China	Susceptible	WT
44	31	9	10	12	10	8	Clinical	India	Resistant	TR$_{34}$/L98H
36	34	11	20	13	9	5	-	France	Susceptible	WT
45	24	10	10	12	4	5	Environmental	India	Susceptible	WT
3	17	9	18	17	3	5	Environmental	Iran	Susceptible	WT
22	26	9	22	13	9	9	Clinical	The Netherlands	Resistant	TR$_{46}$/Y121F/T289A
5	26	9	21	13	10	9	Environmental	Ireland	Resistant	TR$_{46}$/Y121F/T289A
14	25	9	19	14	9	9	Clinical	China	Resistant	TR$_{46}$/Y121F/T289A
13	22	10	26	12	10	10	Clinical	China	Resistant	TR$_{34}$/L98H
46	22	12	23	15	11	8	Environmental	India	Susceptible	WT
96	26	9	40	15	10	7	Environmental	Paraguay	Resistant	WT
72	28	9	35	12	11	10	Environmental	Peru	Susceptible	WT
47	16	7	32	17	15	16	Environmental	Mexico	Resistant	WT
80	18	14	36	19	7	13	Environmental	Peru	Susceptible	WT
79	24	11	36	23	8	9	Environmental	Peru	Susceptible	WT
21	32	25	27	20	10	8	Clinical	The Netherlands	Resistant	TR$_{34}$/L98H
54	17	6	40	5	11	19	Environmental	Mexico	Susceptible	WT
52	21	7	31	7	12	19	Environmental	Mexico	Resistant	TR$_{34}$/L98H
70	17	9	32	14	8	20	Environmental	Peru	Resistant	TR$_{34}$/L98H
67	9	7	32	7	12	19	Environmental	Peru	Resistant	TR$_{34}$/L98H
63	14	8	33	11	9	9	Environmental	Mexico	Susceptible	WT
89	13	17	34	4	6	16	Environmental	Nigeria	Susceptible	WT
74	20	14	27	6	10	18	Environmental	Peru	Susceptible	WT
71	31	8	30	7	6	16	Environmental	Peru	Resistant	TR$_{34}$/L98H
68	27	8	32	6	6	16	Environmental	Peru	Resistant	TR$_{34}$/L98H
66	29	8	33	4	7	23	Environmental	Peru	Resistant	TR$_{34}$/L98H/S297T/F495I
53	44	8	32	7	9	19	Environmental	Mexico	Resistant	TR$_{46}$/Y121F/T289A
51	42	7	32	6	9	19	Environmental	Mexico	Resistant	TR$_{34}$/L98H
49	40	8	31	9	11	19	Environmental	Mexico	Resistant	TR$_{34}$/L98H
69	32	7	30	8	8	19	Environmental	Peru	Resistant	TR$_{34}$/L98H
50	31	10	40	9	9	18	Environmental	Mexico	Resistant	TR$_{34}$/L98H
27	42	9	43	12	10	20	Environmental	Colombia	Susceptible	WT
32	40	9	11	8	10	20	-	Denmark	Resistant	TR$_{34}$/L98H
23	38	10	20	8	10	20	Clinical	The Netherlands	Resistant	TR$_{34}$/L98H
34	43	10	7	8	10	24	-	Denmark	Susceptible	WT
11	31	9	10	8	10	28	Environmental	Romania	Resistant	TR$_{34}$/L98H
4	32	9	6	8	10	20	Environmental	Iran	Resistant	TR$_{34}$/L98H
2	31	9	6	8	10	20	Clinical	Australia	Resistant	TR$_{34}$/L98H
25	29	8	10	9	10	18	Environmental	The Netherlands	Susceptible	WT
48	42	6	9	10	12	15	Environmental	Mexico	Resistant	TR$_{34}$/L98H
38	31	11	10	8	14	20	Environmental	France	Resistant	TR$_{34}$/L98H
37	43	8	11	12	9	20	Environmental	France	Resistant	TR$_{46}$/Y121F/T289A
30	43	8	10	12	9	20	Environmental	Colombia	Resistant	TR$_{46}$/Y121F/T289A
41	42	8	11	12	9	20	Clinical	Germany	Resistant	TR$_{46}$/Y121F/T289A
39	42	9	10	12	10	20	Clinical	France	Resistant	TR$_{46}$/Y121F/T289A

Figure S7. Genetic relationship and characteristics of selected *Aspergillus fumigatus* isolates according to short tandem repeats typing. Characteristics and hierarchical clustering analysis (Euclidean distances by Ward's method) between triazole-resistant and selected susceptible isolates from our study and worldwide isolates describing two clades, clade I and clade II, at a genetic height of 9 (range 0-9.21, mean 3.25). *Scale represents genetic height relatedness.* Abbreviations: Identification (ID).
